# Management of Advanced-stage Hypopharyngeal Carcinoma: 25-Year Experience from a Tertiary Care Medical Center

**DOI:** 10.7759/cureus.6679

**Published:** 2020-01-16

**Authors:** Toms Vengaloor Thomas, Mary R Nittala, Eldrin Bhanat, Ashley A Albert, Srinivasan Vijayakumar

**Affiliations:** 1 Radiation Oncology, University of Mississippi Medical Center, Jackson, USA

**Keywords:** hypopharyngeal carcinoma, advanced-stage head and neck cancer, chemoradiation for hypopharyngeal cancer, definitive surgery, definitive chemoradiation, laryngeal preservation, retrospective review

## Abstract

Introduction

Due to conflicting data in the literature, there is a continuing debate on whether advanced hypopharyngeal carcinoma patients should be treated with definitive surgery or chemoradiotherapy. The purpose of this study is to evaluate the management and outcomes of advanced hypopharyngeal carcinoma in a tertiary care institution over the last 25 years.

Methods

An Institutional Review Board (IRB)-approved and HIPPA-compliant retrospective analysis was performed of patients with advanced-stage squamous cell carcinoma of the hypopharynx treated at our institution between January 1994 and December 2018. Data regarding demographics, stage, treatment, and follow-up were collected. Outcomes including median survival and overall survival were calculated using the Kaplan Meier method. All analyses were performed using SPSS v. 24.

Results

This study included a total of 103 advanced stage hypopharyngeal cancer patients. The median age for this cohort is 61 years (range: 41-88, SD 9.3). Of the total 103 eligible patients treated, 92 (89.3%) were male and 11 (10.7%) female; 61 (59.2%) were African Americans, 39 (37.9%) were Caucasians and three (2.9%) were other races. Seventeen patients (16.5%) had stage III disease, whereas 86 (83.5%) patients were diagnosed with Stage IV A or B disease. Seventy-two patients (69.9%) were treated with definitive chemoradiotherapy (ChemoRT group), and 31 patients (30.1%) underwent primary surgery with or without adjuvant treatments (Surgery group). The two treatment groups were similar in terms of age, gender, ethnicity, alcohol status, N staging, and subsites but were significantly different for smoking status (*p* = 0.035) and T staging (*p *= 0.024). The median follow-up was 17 months. The median survival of the overall cohort was 26 months, and five-year overall survival was 25.5%. The median survival was found to be significantly better for the surgery group as compared to the definitive chemoradiotherapy group (43 months vs 16 months, *p *= 0.049). The five-year overall survival (OS; 41.5% vs 18.5%, *p* = 0.049) and disease-free survival (DFS; 75.3% vs 56%; *p* = 0.029) were significantly better for patients in the surgery group compared to the chemoradiotherapy group. On multivariate Cox-regression analysis, lymph nodal status (HR = 1.27, CI: 1.00-1.62, *p *= 0.047) and chemoradiation treatment (HR = 1.82, CI: 1.00-3.29, *p *= 0.048) were associated with higher risk of mortality.

Conclusion

In our single institutional experience of advanced hypopharyngeal carcinoma management, the five-year overall survival rate was found to be 25.5 % and was the poorest among head and neck cancers. The patients with advanced hypopharyngeal cancer treated with surgery followed by adjuvant radiation or chemoradiation have significantly improved overall survival compared to those treated with definitive chemoradiotherapy. Further research warranted for early detection and better treatment to improve the cure rate in hypopharyngeal carcinoma patients.

## Introduction

Hypopharyngeal carcinoma is a rare malignancy that contributes to approximately 3 to 5% of head and neck carcinomas [1-2]. Approximately 3,400 new cases of hypopharyngeal cancer are diagnosed in the United States every year [3]. The majority of these patients (70% to 85%) present with advanced-stage disease (stage III & IV) [4-6]. Due to the presentation in the advanced stages, hypopharyngeal carcinoma patients tend to have the worst survival among cancers of the head and neck region, with five-year overall survival ranging from 25% to 41% [7-11]. Regarding the treatment options, definitive surgery or laryngeal preservation using chemoradiotherapy is considered the standard of care, but there is a continuing debate on the outcomes due to conflicting data [2,6,10-20]. The purpose of this study is to evaluate the management and outcomes of advanced hypopharyngeal carcinoma in a tertiary care institution in the United States over the past 25 years. 

## Materials and methods

Selection of patients

**Figure 1:**
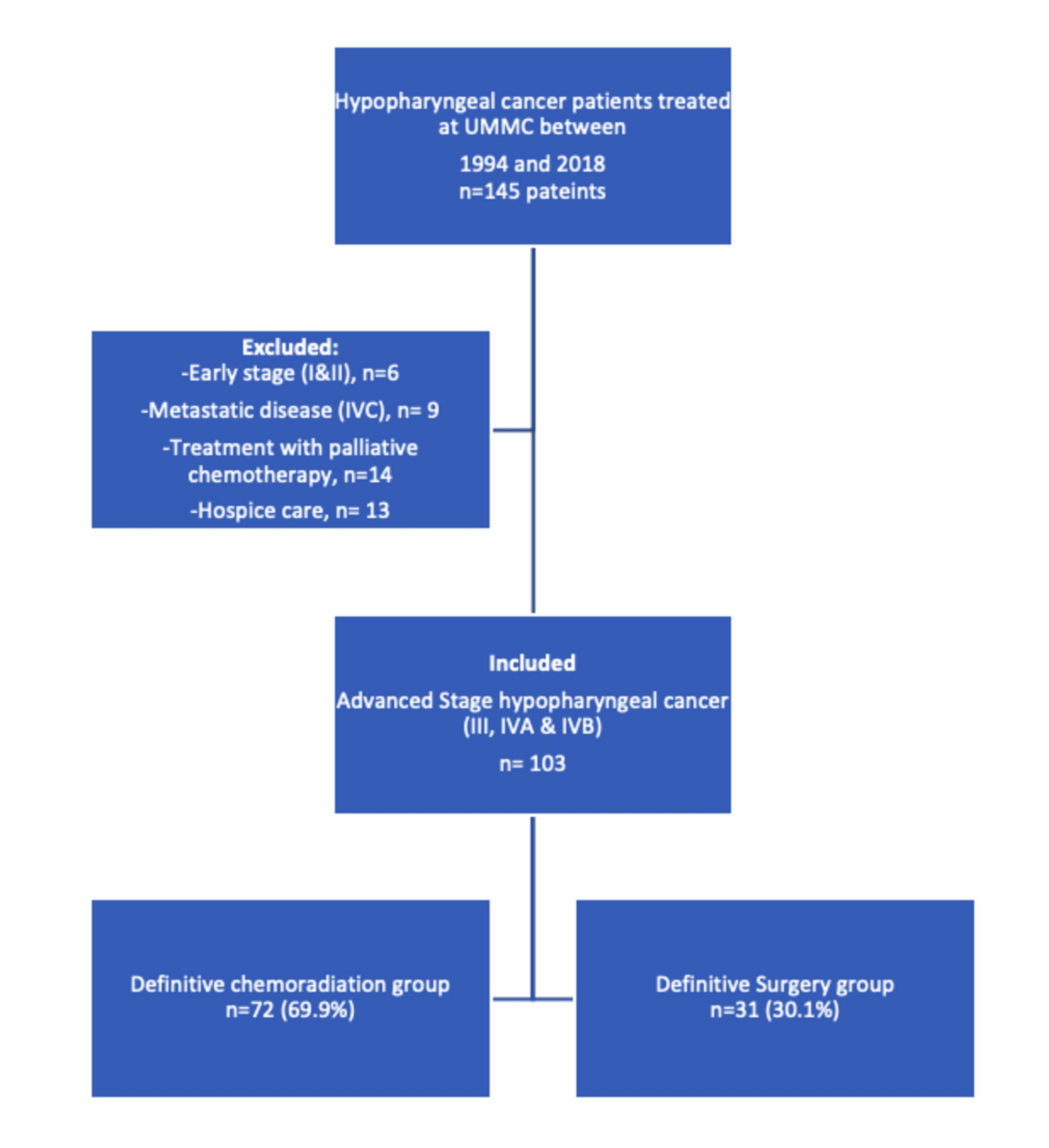
Cohort selection – flow chart for advanced hypopharynx cancer

The Institutional review board (IRB) of the University of Mississippi Medical Center (UMMC) approved all the investigations. The written consent was waived due to the retrospective nature of the study. Data were collected by review of patient charts from the Head and Neck Cancer database of UMMC, diagnosed between January 1994 and December 2018. Research electronic data capture (RedCap), a browser-based database tool, was used to gather and store the patient's information in password-protected computers. We identified 145 patients with hypopharyngeal cancer treated at our institution during the time frame. We collected patient demographic data, including age, sex, race, smoking history, alcohol abuse history, insurance status, and, weight at diagnosis, and tumor characteristics data including pathology, subsite, clinical-stage, the pathological stage. The TNM classification system of the American Joint Committee on Cancer 7th Edition (AJCC 7) was used for staging. The data on management options including surgery, adjuvant radiation, adjuvant chemoradiation, definitive chemoradiation, salvage treatments, palliative chemotherapy, or hospice care were also collected from the patient charts and MOSAIQ record and verify system in radiation oncology. The institutional cancer registry provided the data on follow up and vital statistics of the patients. The exclusion criteria were early-stage disease (stages I or II), metastatic disease at presentation (stage IVC), treatment with palliative intent, or hospice care. One hundred and three patients met the inclusion criteria. We excluded six patients due to early-stage disease, nine patients due to metastatic disease at presentation, and twenty-seven patients due to treatment with palliative intent (Figure 1).

Surgical management

The patients with advanced disease (Stage III, IV A) underwent total laryngectomy and partial pharyngectomy with bilateral or unilateral neck dissection if they were not candidates for laryngeal preservation. Thirty-one (30.1%) patients underwent primary surgical management. Five patients underwent surgery only, and 26 patients underwent adjuvant treatments after definitive surgery.

Radiation treatment and chemotherapy

The radiotherapy was used either as definitive or as adjuvant treatment. Definitive concurrent chemoradiotherapy was delivered as part of laryngeal preservation for advanced stage (III & IV A) or patients with the unresectable disease (Stage IV B). In all, 72 patients (69.9%) underwent definitive chemoradiation to a dose of 7000 cGy. The patients were treated with adjuvant radiation or adjuvant chemoradiation after definitive surgery, depending on risk factors. Eighteen patients received adjuvant radiation treatment, and eight patients underwent adjuvant chemoradiotherapy to a dose of 60-66Gy. The patients had been treated with three-dimensional (3D) treatment planning techniques before 2008 and switched to intensity-modulated radiation treatment (IMRT) technique as IMRT became available in 2008. All of the radiation treatment plans were evaluated by departmental peer review before the initiation of treatment [21-23]. The choice of chemotherapy administered concurrently with radiation was at the discretion of the treating medical oncologist. The most commonly used regimen was weekly cisplatin. Carboplatin or cetuximab was the other systemic therapy options if the patient was not cisplatin eligible due to comorbidities, mainly renal dysfunction.

Statistical analysis

We used SPSS 24.0 software for data analysis. Kaplan-Meier method was used to evaluate overall survival (OS), disease-free survival (DFS), and the log-rank test measured comparison between different treatment groups. The patients who were alive at the time of the last follow up were categorized as censored cases. The multivariate Cox regression model determined the co-variables associated with the OS and DFS. Hazards ratio (HR) was used to estimate time to event outcome with associated 95% confidence intervals (CIs), and *p*-values ≤ 0.05 were considered statistically significant.

## Results

Patient characteristics

This study included a total of 103 advanced stage hypopharyngeal cancer patients. The median age for this cohort is 61 years (range: 41-88, SD: 9.3). Of the total 103 eligible patients treated, 92 (89.3%) were males and 11 (10.7%) were females. Sixty-one (59.2%) patients were African Americans, 39 (37.9%) were Caucasians and, three (2.9%) were other races. Seventeen patients (16.5%) had stage III disease, whereas 86 (83.5%) patients had stage IV A or B disease. Pyriform sinus was the most commonly affected subsite (73%), followed by the posterior pharyngeal wall (10%) and post cricoid area (5%). Seventy-two (69.9%) patients underwent definitive chemoradiotherapy (ChemoRT group), and 31 (30.1%) patients underwent definitive surgery with or without adjuvant treatments (Surgery group). The patient characteristics are summarized in Table 1. The two treatment groups were similar in age, gender, ethnicity, alcohol status, N staging, and subsites but were significantly different for smoking status (*p *= 0.035) and T staging (*p* = 0.024).

**Table 1:** Characteristics of advanced stage (III, IVA & IVB) hypopharyngeal cancer *ChemoRT = definitive chemoradiation therapy; RT = radiation therapy

	All Patients	Definitive ChemoRT	Surgery	p-value
	(n = 103)	n = 72 (69.9%)	n = 31 (30.1%)	
Age, Mean (SD)	60.3 (9.3)	59.7 (9.5)	61.2 (8.8)	0.508
Gender/ Sex				
Male	92 (89.3%)	64 (89.9%)	28 (90.3%)	0.829
Female	11 (10.7%)	8 (11.1%)	3 (9.7%)	
Ethnicity				
Caucasians	39 (37.9%)	28 (38.9%)	11 (35.5%)	0.945
African Americans	61 (59.2%)	42 (58.3%)	19 (61.3%	
Others	3 (2.9%)	2 (2.8%)	1 (3.2%)	
Smoking status				0.035
Smoker	80 (77.7%)	60 (83.3%)	20 (64.5%)	
Non-Smoker	23 (22.3%)	12 (16.7%)	11 (35.5%)	
Alcohol Status				0.98
Drinker	60 (58.3%)	42 (58.3%)	18 (58.1%)	
Non- Drinker	43 (41.7%)	30 (41.7%)	13 (41.9%)	
TNM Stage (III, IV)				
T classification				0.024
T1	8 (7.8%)	5 (6.9%)	3 (9.7%)	
T2	13 (12.6%)	12 (16.7%)	1 (3.2%)	
T3	17 (16.5%)	8 (11.1%)	9 (29.0%)	
T4	41 (39.8%)	33 (45.8%)	8 (25.8%)	
Unknown	24 (23.3%)	14 (19.4%)	10 (32.3%)	
N Classification				0.116
N0	20 (19.4%)	12 (16.7%)	8 (25.8%)	
N1	8 (7.8%)	7 (9.7%)	1 (3.2%)	
N2	45 (43.7%)	34 (47.2%)	11 (35.5%)	
N3	9 (8.7%)	8 (11.1%)	1 (3.2%)	
Unknown	21 (20.4%)	11 (15.3%)	10 (32.3%)	
Sub-site				0.057
Pyriform Sinus	75 (72.8%)	51 (70.8%)	24 (77.4%)	
Post-pharyngeal Wall	10 (9.7%)	10 (13.9%)	0 (0.0%)	
Post-Cricoid	5 (4.9%)	3 (4.2%)	2 (6.5%)	
Hypopharynx Unknown	13 (12.3%)	8 (11.2 %)	5 (16.1 %)	

**Figure 2:**
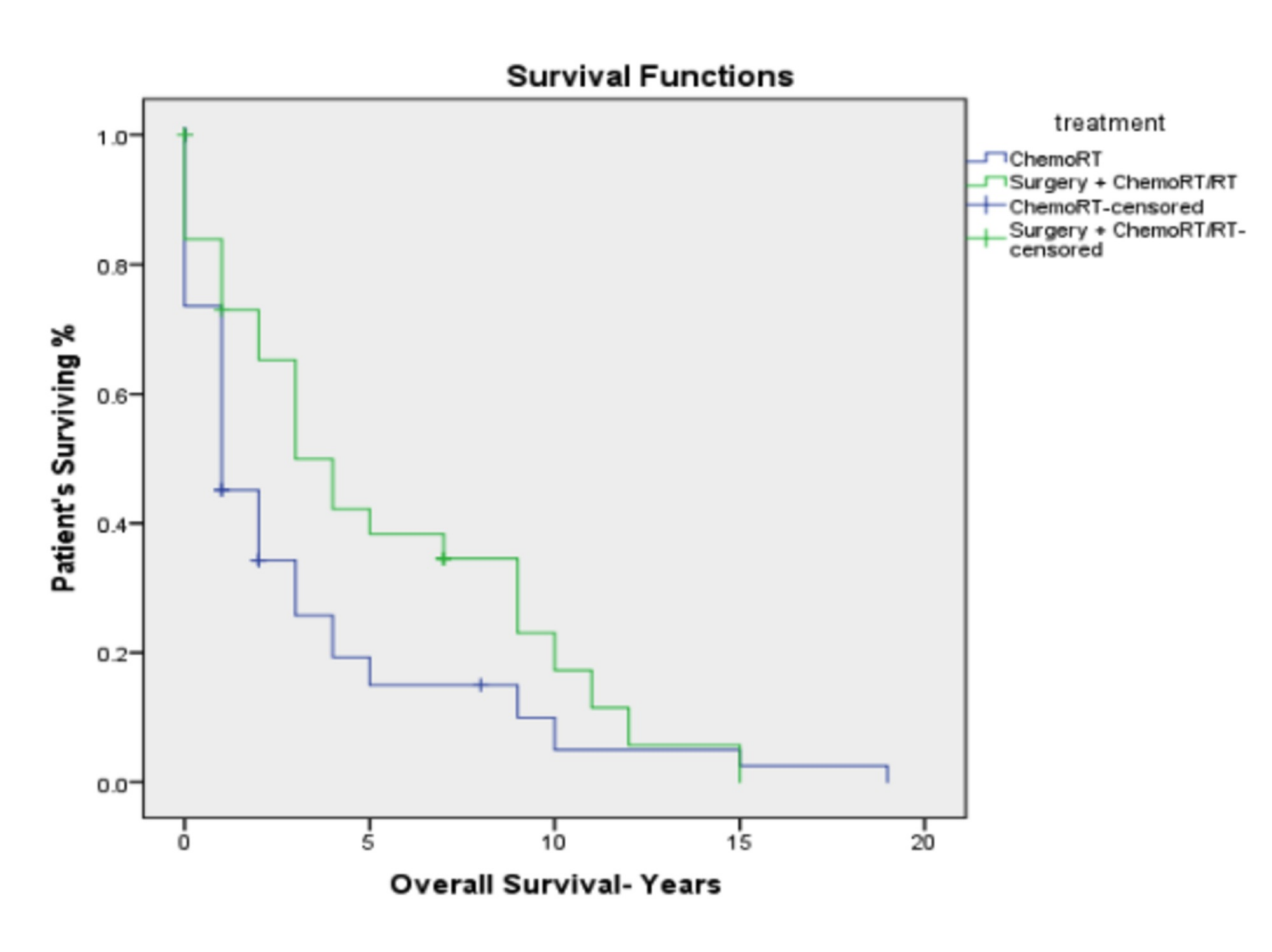
Kaplan–Meier overall survival for advanced stage hypopharyngeal cancer by treatment modality

**Figure 3:**
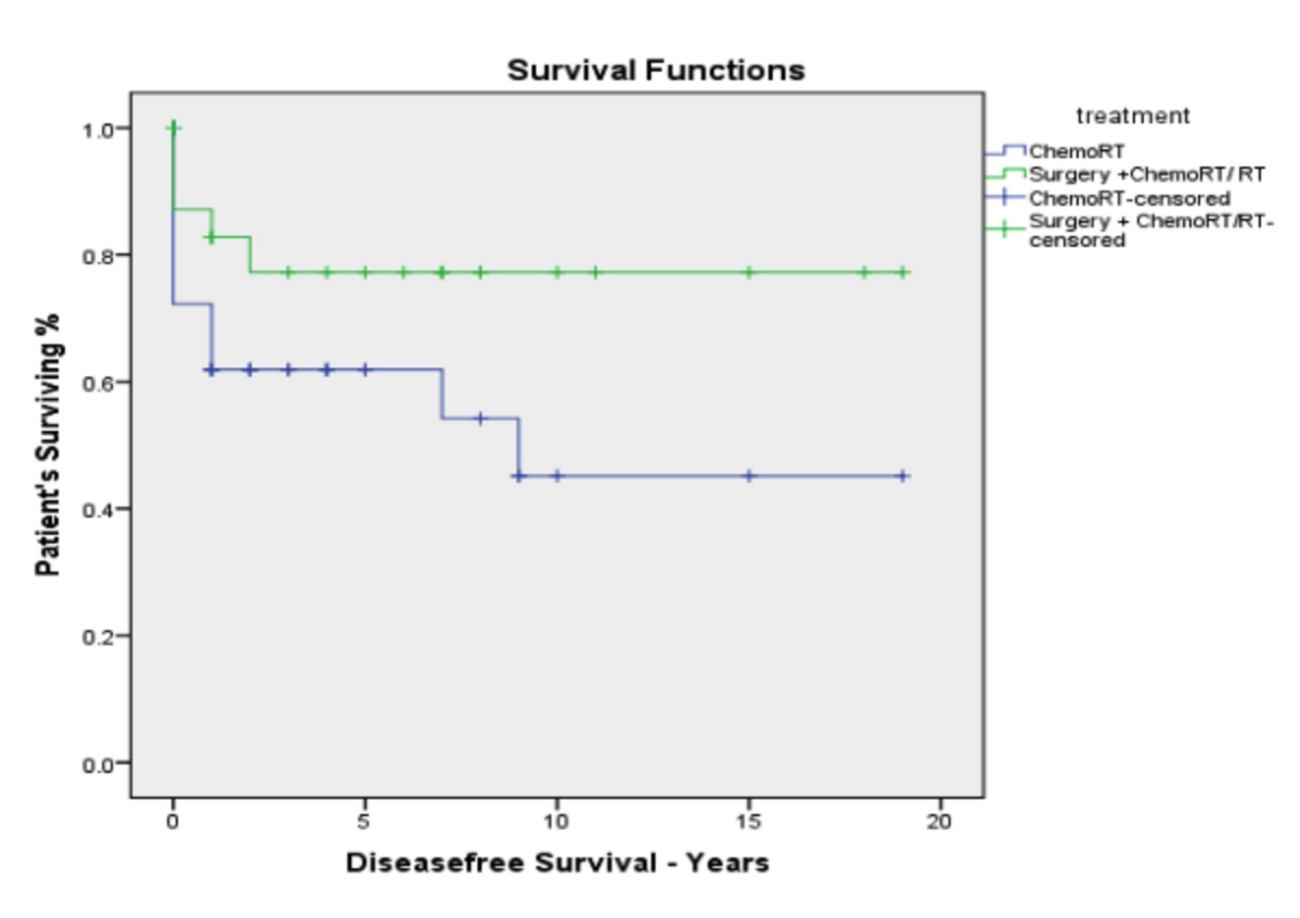
Kaplan–Meier disease-free survival for advanced stage hypopharyngeal carcinoma by treatment

The median follow-up was 17 months. The whole cohort of patients had a median survival of 26 months and a five-year overall survival of 25.5%. The median survival was found to be significantly better for the surgery group as compared to the definitive chemoradiotherapy group (43 months vs. 16 months, *p *= 0.049). The five-year OS was found to be significantly better for patients in the surgery group compared to the chemoradiotherapy group (41.5% vs. 18.5%, *p *= 0.049; Figure 2). The Corresponding five-year disease-free survival (DFS) was also better for the surgery group (75.3% vs 56%; *p *= 0.029; Figure 3). There was no difference in five-year survival between the patients treated with 3D CRT compared to those treated with IMRT. 

Multivariate Cox-regression analysis (MVA) was performed to identify the independent predictors of OS and assess potential confounding variables (Table 2). MVA revealed that chemoradiation treatment (HR = 1.82, CI: 1.00-3.29, *p* = 0.048) and lymph nodal status (HR = 1.27, CI: 1.00-1.62, *p* = 0.047) were associated with an increased risk of mortality. Variables including age, gender, ethnicity, smoking status, T staging, and subsites were not predictors for survival. Only nodal status was found to be a predictor of DFS (HR = 1.80, CI: 1.13-2.86, *p* = 0.013) on multivariate analysis. 

**Table 2:** Multivariable Cox Regression for OS and DFS *ChemoRT = definitive chemoradiation therapy; RT = radiation therapy; HR = hazard ratio; CI = confidence interval; OS = overall survival; DFS = disease-free survival

	Overall Survival (OS)		Disease-free Survival (DFS)	
	HR (95% CI)	P-value	HR (95% CI)	P-Value
Gender/Sex	1.47 (0.60-3.58)	0.387	2.59 (0.71-9.41)	0.147
Age	0.67 (0.34-1.32)	0.251	0.54 (0.23-1.27)	0.164
Ethnicity	1.21 (0.71-2.03)	0.472	0.84 (0.38-1.86)	0.671
Smoking Status	1.11 (0.45-2.70)	0.818	0.31 (0.70-1.45)	0.140
Alcohol Status	0.82 (0.45-1.50)	0.530	0.82 (0.36-1.86)	0.636
Overall Stage III vs IV	0.93(0.42-2.07)	0.867	0.24 (0.02-2.02)	0.191
T classification	1.12 (0.93-1.34)	0.218	1.13 (0.84-1.51)	0.393
N classification	1.27 (1.00-1.62)	0.047	1.80 (1.13-2.86)	0.013
Subsites	0.95 (0.40-2.22)	0.908	0.82 (0.59-1.14)	0.243
Treatment	1.82 (1.00–3.29)	0.048	1.63 (0.64-4.14)	0.297

## Discussion

Hypopharyngeal cancer is a rare malignancy of the head and neck region, contributing to only 3% to 5% of all the head and neck cancers [1-2]. Our data is consistent with the literature, with hypopharyngeal cancer constituting 4% of all the patients in the head and neck cancer database of our institution. Patients with hypopharyngeal squamous cell carcinoma are mostly males with a known history of tobacco (90%) and heavy alcohol use (50%) [18].

The National Cancer Database (NCDB) data shows that patients with hypopharyngeal cancer are, on average, 63 years old, 75% male, and Caucasian over 70% of times. In contrast to this, our patient cohort consisted of mainly African American patients (59.2%), likely due to the demographics of the region. In our patient cohort, 96% of the patients presented with advanced-stage disease (stage III & IV), as compared to 70% to 85% reported in the literature [4-6]. This difference is likely due to disparities in access to care. 

Hypopharyngeal cancers usually have a locally aggressive pattern and are reported to have the worst prognosis among all the head and neck cancer sites [4]. The patients with advanced-stage hypopharyngeal cancer have five-year OS, ranging from 25% to 41% [21]. There are few single-institution experiences where they report a higher five-year overall survival of 62%and 66% [12,16]. Compared to this data, our patients had a median survival of 26 months and the five-year overall survival of 25.5%, which is consistent with the majority of literature. 

Historically, surgery followed by adjuvant radiation has been the standard-of-care for patients presenting with advanced disease. The European Organization for Research and Treatment of Cancer (EORTC) 24891 trial proved that laryngeal preservation using chemoradiation treatment is an equally effective treatment, thus became the standard of care [19-20]. Another randomized control trial by Beauvillain et al. reported a contradicting result in which they found improved five-year overall survival (37% vs. 19%, *p *= 0.04) and local control (63% vs. 39%, *p *= 0.01) in the surgery arm [6]. Of note, both these trials used induction chemotherapy followed by radiation treatment as the strategy for laryngeal preservation, instead of concurrent chemoradiotherapy. Retrospective reviews have also reported conflicting results. A retrospective single-institution review by Harris et al. identified a clinical improvement in five-year survival with the use of surgery followed by adjuvant treatments over definitive concurrent chemoradiation treatment but was not statistically significant (66.3% vs. 41.3%, *p *= 0.09) [12]. A population-based experience from the Netherlands reported that surgical management and laryngeal preservation treatment had equivalent survival for patients with T3 disease (40% vs. 39%, *p *= 0.475), but not for patients with T4 disease (29% vs. 24%, *p *= 0.039) [22]. Contrary to this, Hall et al., Lee et al., Zelefsky et al., Iwae et al., and Lajtman et al. reported an equivalent survival outcome between surgical or non-surgical management for advanced hypopharyngeal cancer [13-15,17,23]. SEER (Surveillance, Epidemiology and, End Results) database analysis did not find a difference in three-year survival for T4 patients between surgical and non-surgical treatments (29.9% vs. 26.1%, *p *= 0.439) [24]. A single-institution experience from Taiwan reported equivalent survival among patients who received laryngeal preservation or operative management [25]. Two systematic reviews have compiled the data on laryngeal preservation for hypopharyngeal cancer and reported that laryngeal preservation offered equivalent survival as surgical treatments [9-10]. In our study, we found that surgical management resulted in an improved five-year overall survival as compared to chemoradiotherapy (41.5% vs. 18.5%, *p *= 0.049). This difference is likely due to the inclusion of laryngeal preservation patients and unresectable patients together in the chemoradiotherapy group. 

Limitations of our study include its retrospective nature and a modest number of patients, which leads to selection bias in the patient cohorts. We were not able to account for the confounding factors, including medical comorbidities, and that might have contributed to the observed survival differences. These patients are treated over the last 25 years, during which radiation techniques, surgical techniques, and systemic therapy have evolved. These changes might be contributing to the results that we observed. Some of the follow-up data are missing, including salvage treatments and causes of death. So, the differences in the survival between the two different treatment groups should be interpreted within the context of the limitations of a non-randomized retrospective study. 

## Conclusions

In our institutional experience over the last 25 years, the majority of the hypopharyngeal carcinoma patients presented with advanced-stage disease. The five-year overall survival rates of our patient cohort were around 25%, which is consistent with the literature. From our analysis, surgical management was found to have better survival as compared to definitive chemoradiotherapy. Further research was warranted for early detection and better treatment to improve the cure rate in hypopharyngeal carcinoma patients.
